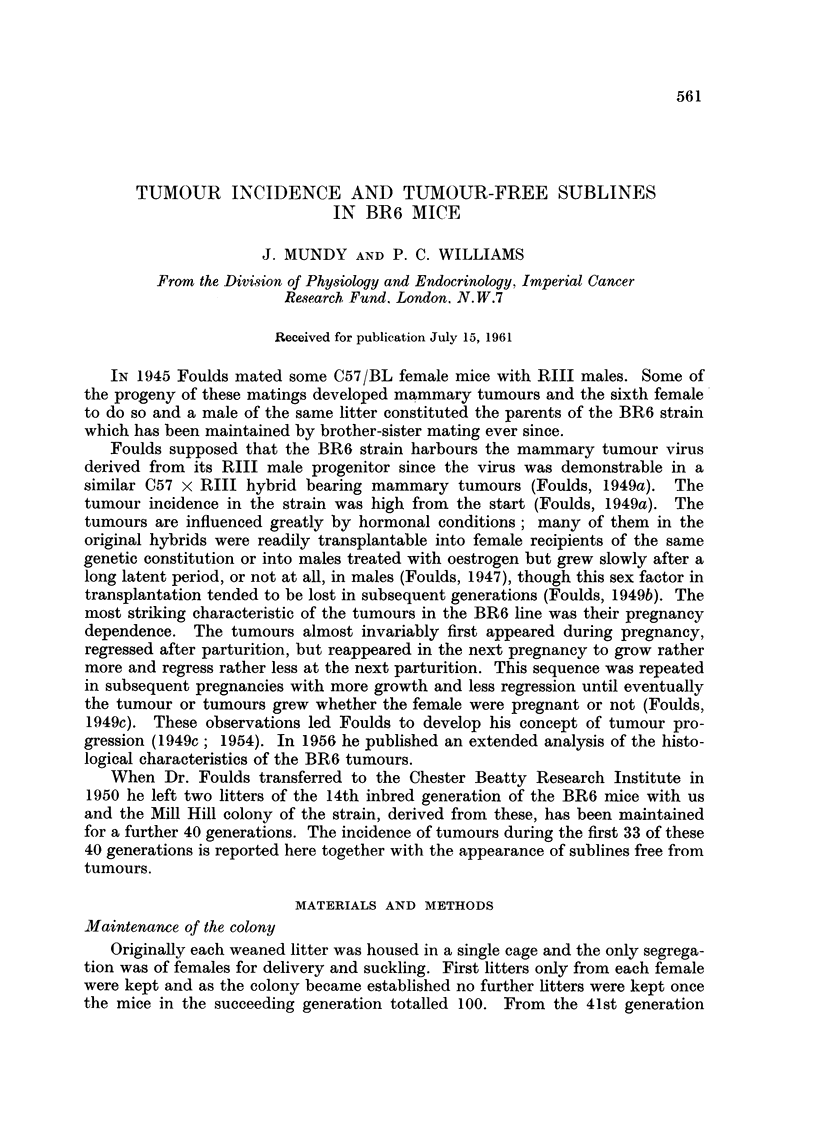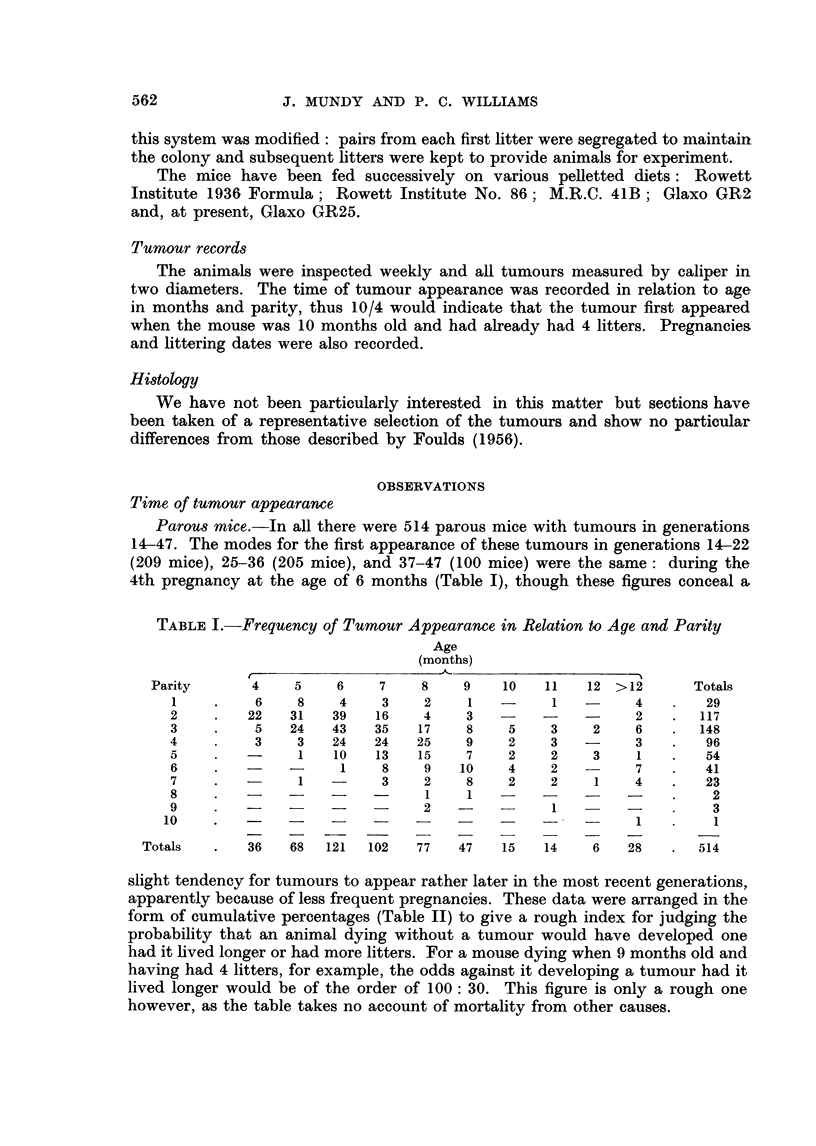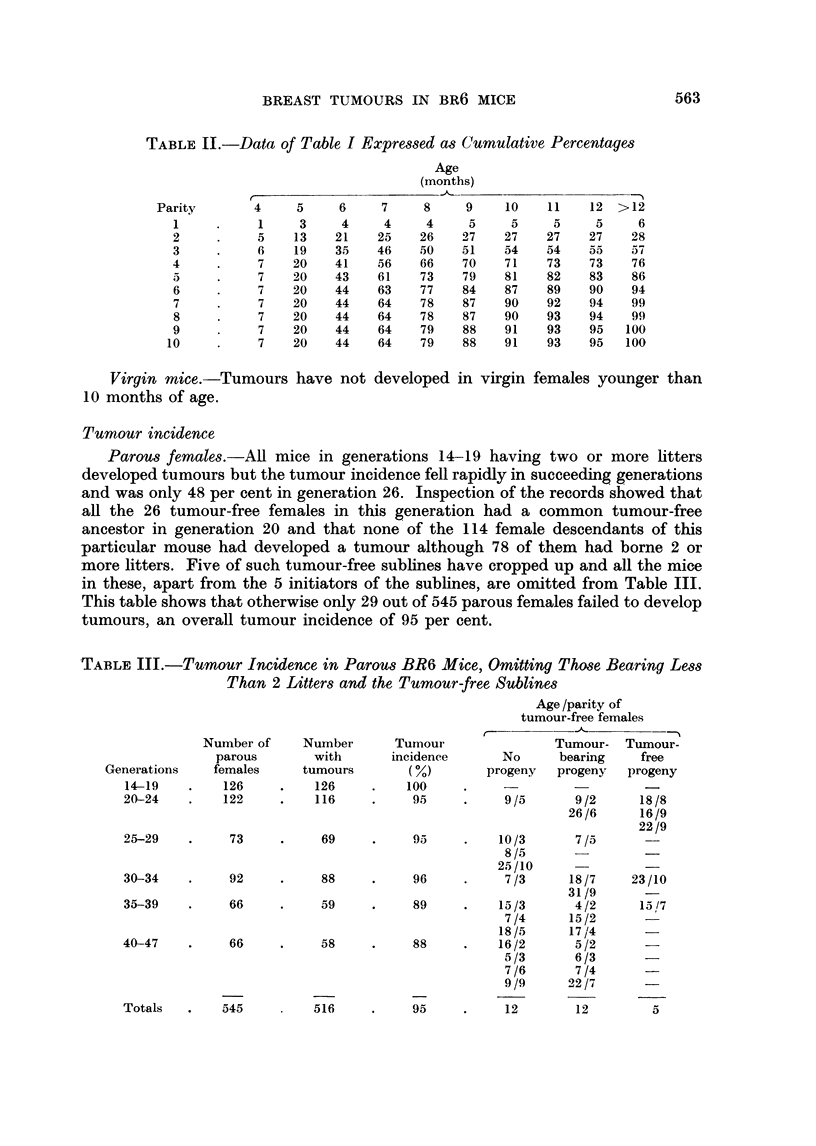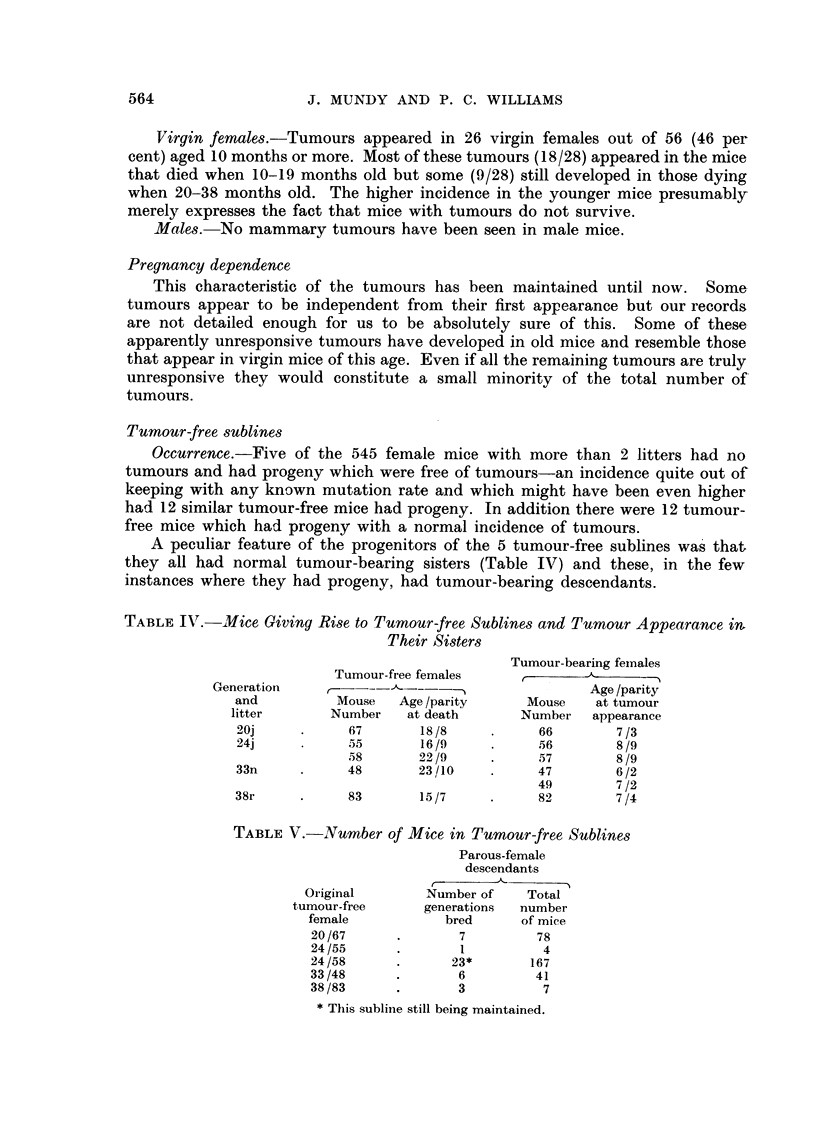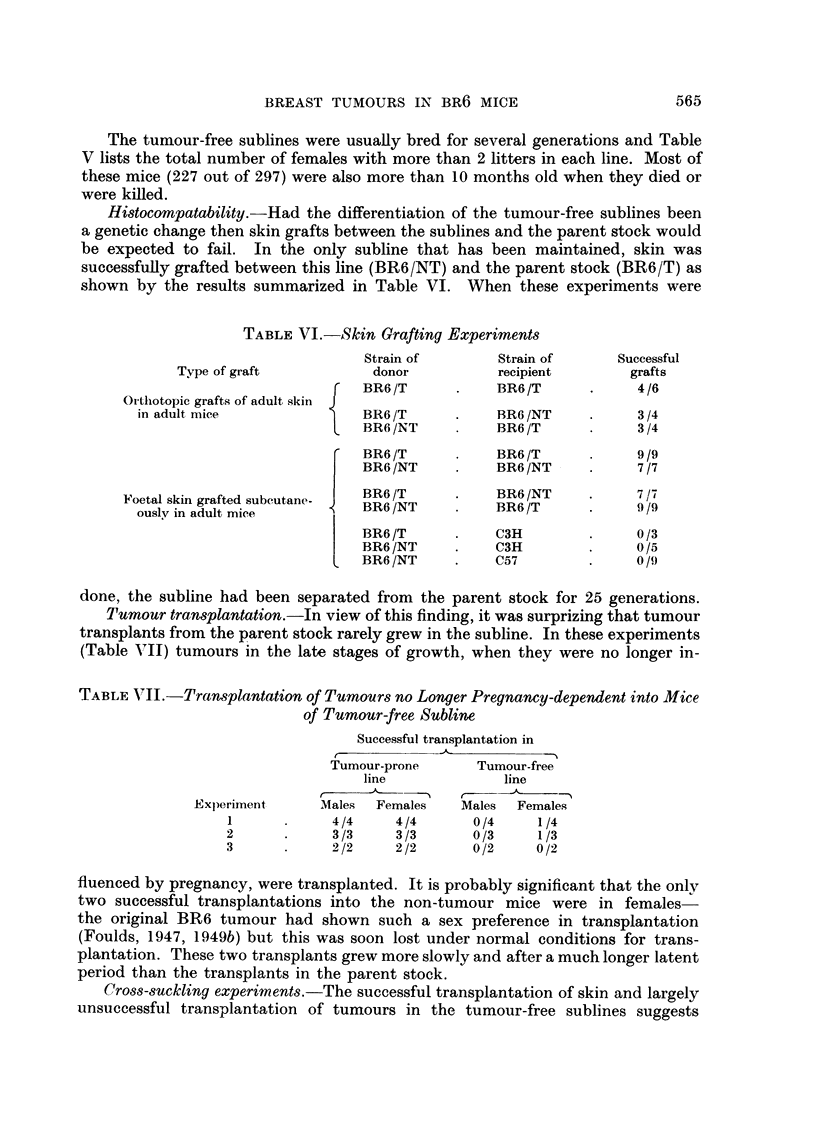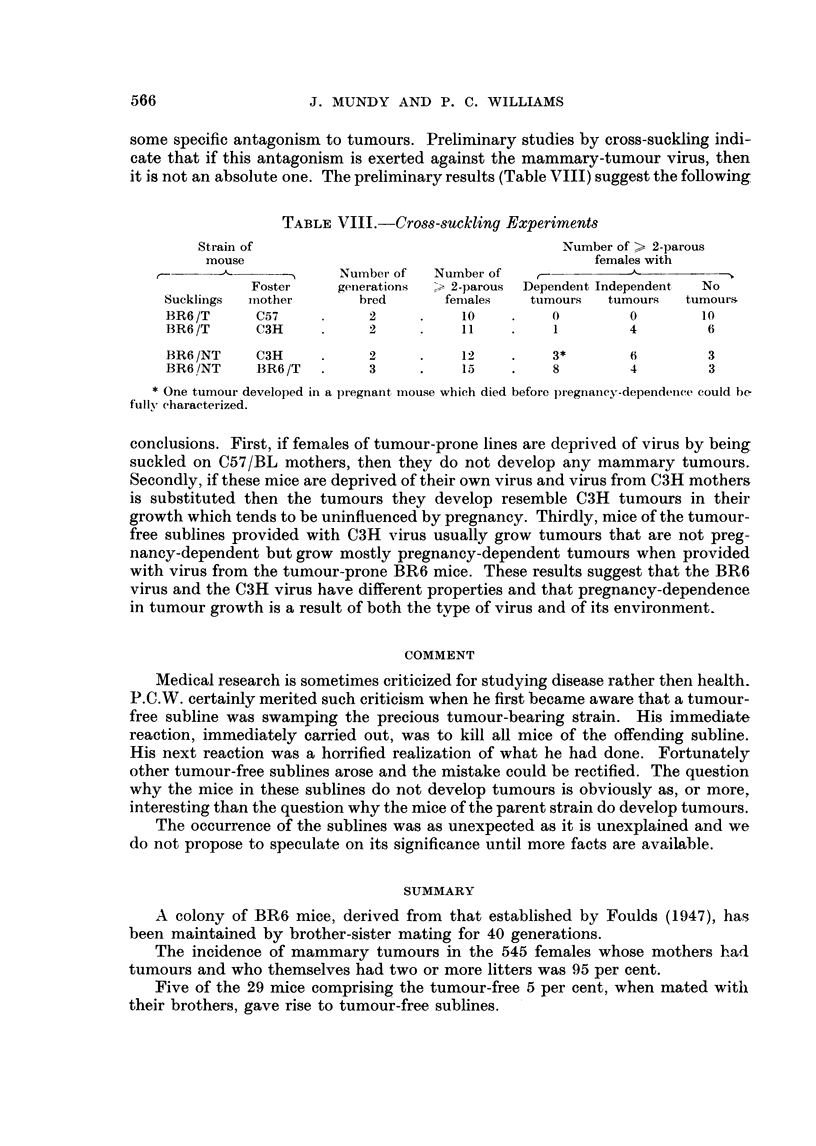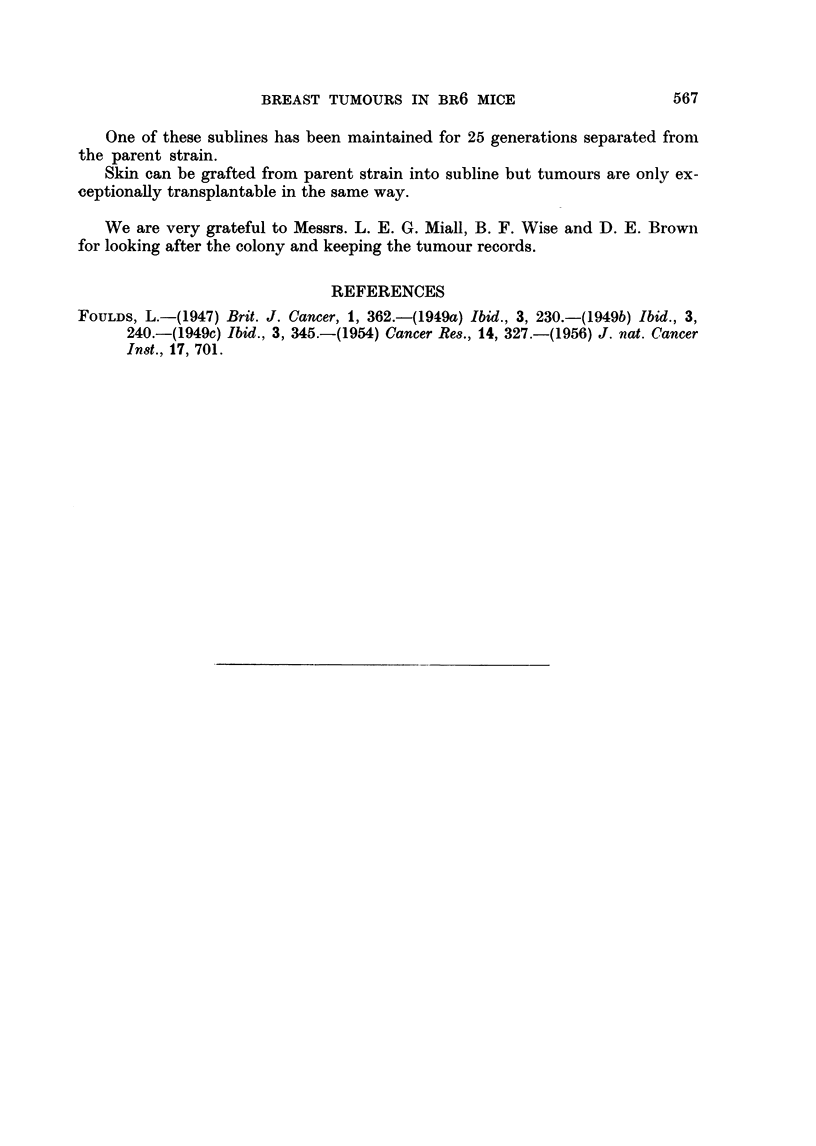# Tumour Incidence and Tumour-Free Sublines in BR6 Mice

**DOI:** 10.1038/bjc.1961.65

**Published:** 1961-09

**Authors:** J. Mundy, P. C. Williams


### TUMOUR INCIDENCE AND TUMOUR-FREE SUBLINES IN BR6 MICE

J. MUNDY AND P. C. WILLIAMS

*From the Division of Physiology and Endocrinology, Imperial Cancer Research Fund, London, N.W.7*

Received for publication July 15, 1961

IN 1945 Foulds mated some C57/BL female mice with RIII males. Some of the progeny of these matings developed mammary tumours and the sixth female to do so and a male of the same litter constituted the parents of the BR6 strain which has been maintained by brother-sister mating ever since.

Foulds supposed that the BR6 strain harbours the mammary tumour virus derived from its RIII male progenitor since the virus was demonstrable in a similar C57 × RIII hybrid bearing mammary tumours (Foulds, 1949*a*). The tumour incidence in the strain was high from the start (Foulds, 1949*a*). The tumours are influenced greatly by hormonal conditions ; many of them in the original hybrids were readily transplantable into female recipients of the same genetic constitution or into males treated with oestrogen but grew slowly after a long latent period, or not at all, in males (Foulds, 1947), though this sex factor in transplantation tended to be lost in subsequent generations (Foulds, 1949*b*). The most striking characteristic of the tumours in the BR6 line was their pregnancy dependence. The tumours almost invariably first appeared during pregnancy, regressed after parturition, but reappeared in the next pregnancy to grow rather more and regress rather less at the next parturition. This sequence was repeated in subsequent pregnancies with more growth and less regression until eventually the tumour or tumours grew whether the female were pregnant or not (Foulds, 1949*c*). These observations led Foulds to develop his concept of tumour progression (1949*c* ; 1954). In 1956 he published an extended analysis of the histological characteristics of the BR6 tumours.

When Dr. Foulds transferred to the Chester Beatty Research Institute in 1950 he left two litters of the 14th inbred generation of the BR6 mice with us and the Mill Hill colony of the strain, derived from these, has been maintained for a further 40 generations. The incidence of tumours during the first 33 of these 40 generations is reported here together with the appearance of sublines free from tumours.

### MATERIALS AND METHODS

### *Maintenance of the colony*

Originally each weaned litter was housed in a single cage and the only segregation was of females for delivery and suckling. First litters only from each female were kept and as the colony became established no further litters were kept once the mice in the succeeding generation totalled 100. From the 41st generation this system was modified : pairs from each first litter were segregated to maintain the colony and subsequent litters were kept to provide animals for experiment.

The mice have been fed successively on various pelletted diets : Rowett Institute 1936 Formula ; Rowett Institute No. 86 ; M.R.C. 41B ; Glaxo GR2 and, at present, Glaxo GR25.

*Tumour records*

The animals were inspected weekly and all tumours measured by caliper in two diameters. The time of tumour appearance was recorded in relation to age in months and parity, thus 10/4 would indicate that the tumour first appeared when the mouse was 10 months old and had already had 4 litters. Pregnancies and littering dates were also recorded.

*Histology*

We have not been particularly interested in this matter but sections have been taken of a representative selection of the tumours and show no particular differences from those described by Foulds (1956).

### OBSERVATIONS

*Time of tumour appearance*

*Parous mice.*—In all there were 514 parous mice with tumours in generations 14–47. The modes for the first appearance of these tumours in generations 14–22 (209 mice), 25–36 (205 mice), and 37–47 (100 mice) were the same : during the 4th pregnancy at the age of 6 months (Table I), though these figures conceal a

TABLE I.—*Frequency of Tumour Appearance in Relation to Age and Parity*

| Parity | Age (months) | | | | | | | | | | Totals |
|---|---|---|---|---|---|---|---|---|---|---|---|
| | 4 | 5 | 6 | 7 | 8 | 9 | 10 | 11 | 12 | >12 | |
| 1 | 6 | 8 | 4 | 3 | 2 | 1 | — | 1 | — | 4 | 29 |
| 2 | 22 | 31 | 39 | 16 | 4 | 3 | — | — | — | 2 | 117 |
| 3 | 5 | 24 | 43 | 35 | 17 | 8 | 5 | 3 | 2 | 6 | 148 |
| 4 | 3 | 3 | 24 | 24 | 25 | 9 | 2 | 3 | — | 3 | 96 |
| 5 | — | 1 | 10 | 13 | 15 | 7 | 2 | 2 | 3 | 1 | 54 |
| 6 | — | — | 1 | 8 | 9 | 10 | 4 | 2 | — | 7 | 41 |
| 7 | — | 1 | — | 3 | 2 | 8 | 2 | 2 | 1 | 4 | 23 |
| 8 | — | — | — | — | 1 | 1 | — | — | — | — | 2 |
| 9 | — | — | — | — | 2 | — | — | 1 | — | — | 3 |
| 10 | — | — | — | — | — | — | — | — | — | 1 | 1 |
| Totals | 36 | 68 | 121 | 102 | 77 | 47 | 15 | 14 | 6 | 28 | 514 |

slight tendency for tumours to appear rather later in the most recent generations, apparently because of less frequent pregnancies. These data were arranged in the form of cumulative percentages (Table II) to give a rough index for judging the probability that an animal dying without a tumour would have developed one had it lived longer or had more litters. For a mouse dying when 9 months old and having had 4 litters, for example, the odds against it developing a tumour had it lived longer would be of the order of 100 : 30. This figure is only a rough one however, as the table takes no account of mortality from other causes.

TABLE II.—*Data of Table I Expressed as Cumulative Percentages*

| Parity | Age (months) 4 | 5 | 6 | 7 | 8 | 9 | 10 | 11 | 12 | >12 |
|---|---|---|---|---|---|---|---|---|---|---|
| 1 | 1 | 3 | 4 | 4 | 4 | 5 | 5 | 5 | 5 | 6 |
| 2 | 5 | 13 | 21 | 25 | 26 | 27 | 27 | 27 | 27 | 28 |
| 3 | 6 | 19 | 35 | 46 | 50 | 51 | 54 | 54 | 55 | 57 |
| 4 | 7 | 20 | 41 | 56 | 66 | 70 | 71 | 73 | 73 | 76 |
| 5 | 7 | 20 | 43 | 61 | 73 | 79 | 81 | 82 | 83 | 86 |
| 6 | 7 | 20 | 44 | 63 | 77 | 84 | 87 | 89 | 90 | 94 |
| 7 | 7 | 20 | 44 | 64 | 78 | 87 | 90 | 92 | 94 | 99 |
| 8 | 7 | 20 | 44 | 64 | 78 | 87 | 90 | 93 | 94 | 99 |
| 9 | 7 | 20 | 44 | 64 | 79 | 88 | 91 | 93 | 95 | 100 |
| 10 | 7 | 20 | 44 | 64 | 79 | 88 | 91 | 93 | 95 | 100 |

*Virgin mice.*—Tumours have not developed in virgin females younger than 10 months of age.

*Tumour incidence*

*Parous females.*—All mice in generations 14–19 having two or more litters developed tumours but the tumour incidence fell rapidly in succeeding generations and was only 48 per cent in generation 26. Inspection of the records showed that all the 26 tumour-free females in this generation had a common tumour-free ancestor in generation 20 and that none of the 114 female descendants of this particular mouse had developed a tumour although 78 of them had borne 2 or more litters. Five of such tumour-free sublines have cropped up and all the mice in these, apart from the 5 initiators of the sublines, are omitted from Table III. This table shows that otherwise only 29 out of 545 parous females failed to develop tumours, an overall tumour incidence of 95 per cent.

TABLE III.—*Tumour Incidence in Parous BR6 Mice, Omitting Those Bearing Less Than 2 Litters and the Tumour-free Sublines*

| | | | | Age/parity of tumour-free females | | |
|---|---|---|---|---|---|---|
| Generations | Number of parous females | Number with tumours | Tumour incidence (%) | No progeny | Tumour-bearing progeny | Tumour-free progeny |
| 14–19 | 126 | 126 | 100 | — | — | — |
| 20–24 | 122 | 116 | 95 | 9/5 | 9/2 | 18/8 |
| | | | | | 26/6 | 16/9 |
| | | | | | | 22/9 |
| 25–29 | 73 | 69 | 95 | 10/3 | 7/5 | — |
| | | | | 8/5 | — | — |
| | | | | 25/10 | — | — |
| 30–34 | 92 | 88 | 96 | 7/3 | 18/7 | 23/10 |
| | | | | | 31/9 | — |
| 35–39 | 66 | 59 | 89 | 15/3 | 4/2 | 15/7 |
| | | | | 7/4 | 15/2 | — |
| | | | | 18/5 | 17/4 | — |
| 40–47 | 66 | 58 | 88 | 16/2 | 5/2 | — |
| | | | | 5/3 | 6/3 | — |
| | | | | 7/6 | 7/4 | — |
| | | | | 9/9 | 22/7 | — |
| Totals | 545 | 516 | 95 | 12 | 12 | 5 |

*Virgin females.*—Tumours appeared in 26 virgin females out of 56 (46 per cent) aged 10 months or more. Most of these tumours (18/28) appeared in the mice that died when 10–19 months old but some (9/28) still developed in those dying when 20–38 months old. The higher incidence in the younger mice presumably merely expresses the fact that mice with tumours do not survive.

*Males.*—No mammary tumours have been seen in male mice.

*Pregnancy dependence*

This characteristic of the tumours has been maintained until now. Some tumours appear to be independent from their first appearance but our records are not detailed enough for us to be absolutely sure of this. Some of these apparently unresponsive tumours have developed in old mice and resemble those that appear in virgin mice of this age. Even if all the remaining tumours are truly unresponsive they would constitute a small minority of the total number of tumours.

*Tumour-free sublines*

*Occurrence.*—Five of the 545 female mice with more than 2 litters had no tumours and had progeny which were free of tumours—an incidence quite out of keeping with any known mutation rate and which might have been even higher had 12 similar tumour-free mice had progeny. In addition there were 12 tumour-free mice which had progeny with a normal incidence of tumours.

A peculiar feature of the progenitors of the 5 tumour-free sublines was that they all had normal tumour-bearing sisters (Table IV) and these, in the few instances where they had progeny, had tumour-bearing descendants.

TABLE IV.—*Mice Giving Rise to Tumour-free Sublines and Tumour Appearance in Their Sisters*

| Generation and litter | Tumour-free females: Mouse Number | Tumour-free females: Age/parity at death | Tumour-bearing females: Mouse Number | Tumour-bearing females: Age/parity at tumour appearance |
|---|---|---|---|---|
| 20j | 67 | 18/8 | 66 | 7/3 |
| 24j | 55 | 16/9 | 56 | 8/9 |
| | 58 | 22/9 | 57 | 8/9 |
| 33n | 48 | 23/10 | 47 | 6/2 |
| | | | 49 | 7/2 |
| 38r | 83 | 15/7 | 82 | 7/4 |

TABLE V.—*Number of Mice in Tumour-free Sublines*

| Original tumour-free female | Parous-female descendants: Number of generations bred | Parous-female descendants: Total number of mice |
|---|---|---|
| 20/67 | 7 | 78 |
| 24/55 | 1 | 4 |
| 24/58 | 23* | 167 |
| 33/48 | 6 | 41 |
| 38/83 | 3 | 7 |

* This subline still being maintained.

The tumour-free sublines were usually bred for several generations and Table V lists the total number of females with more than 2 litters in each line. Most of these mice (227 out of 297) were also more than 10 months old when they died or were killed.

*Histocompatability.*—Had the differentiation of the tumour-free sublines been a genetic change then skin grafts between the sublines and the parent stock would be expected to fail. In the only subline that has been maintained, skin was successfully grafted between this line (BR6/NT) and the parent stock (BR6/T) as shown by the results summarized in Table VI. When these experiments were done, the subline had been separated from the parent stock for 25 generations.

TABLE VI.—*Skin Grafting Experiments*

| Type of graft | Strain of donor | Strain of recipient | Successful grafts |
|---|---|---|---|
| Orthotopic grafts of adult skin in adult mice | BR6/T | BR6/T | 4/6 |
| | BR6/T | BR6/NT | 3/4 |
| | BR6/NT | BR6/T | 3/4 |
| Foetal skin grafted subcutaneously in adult mice | BR6/T | BR6/T | 9/9 |
| | BR6/NT | BR6/NT | 7/7 |
| | BR6/T | BR6/NT | 7/7 |
| | BR6/NT | BR6/T | 9/9 |
| | BR6/T | C3H | 0/3 |
| | BR6/NT | C3H | 0/5 |
| | BR6/NT | C57 | 0/9 |

*Tumour transplantation.*—In view of this finding, it was surprizing that tumour transplants from the parent stock rarely grew in the subline. In these experiments (Table VII) tumours in the late stages of growth, when they were no longer influenced by pregnancy, were transplanted. It is probably significant that the only two successful transplantations into the non-tumour mice were in females—the original BR6 tumour had shown such a sex preference in transplantation (Foulds, 1947, 1949*b*) but this was soon lost under normal conditions for transplantation. These two transplants grew more slowly and after a much longer latent period than the transplants in the parent stock.

TABLE VII.—*Transplantation of Tumours no Longer Pregnancy-dependent into Mice of Tumour-free Subline*

| | Successful transplantation in | | | |
|---|---|---|---|---|
| | Tumour-prone line | | Tumour-free line | |
| Experiment | Males | Females | Males | Females |
| 1 | 4/4 | 4/4 | 0/4 | 1/4 |
| 2 | 3/3 | 3/3 | 0/3 | 1/3 |
| 3 | 2/2 | 2/2 | 0/2 | 0/2 |

*Cross-suckling experiments.*—The successful transplantation of skin and largely unsuccessful transplantation of tumours in the tumour-free sublines suggests some specific antagonism to tumours. Preliminary studies by cross-suckling indicate that if this antagonism is exerted against the mammary-tumour virus, then it is not an absolute one. The preliminary results (Table VIII) suggest the following

TABLE VIII.—*Cross-suckling Experiments*

| Strain of mouse | | | | Number of $\geqslant$ 2-parous females with | | |
|---|---|---|---|---|---|---|
| Sucklings | Foster mother | Number of generations bred | Number of $\geqslant$ 2-parous females | Dependent tumours | Independent tumours | No tumours |
| BR6/T | C57 | 2 | 10 | 0 | 0 | 10 |
| BR6/T | C3H | 2 | 11 | 1 | 4 | 6 |
| BR6/NT | C3H | 2 | 12 | 3* | 6 | 3 |
| BR6/NT | BR6/T | 3 | 15 | 8 | 4 | 3 |

* One tumour developed in a pregnant mouse which died before pregnancy-dependence could be fully characterized.

conclusions. First, if females of tumour-prone lines are deprived of virus by being suckled on C57/BL mothers, then they do not develop any mammary tumours. Secondly, if these mice are deprived of their own virus and virus from C3H mothers is substituted then the tumours they develop resemble C3H tumours in their growth which tends to be uninfluenced by pregnancy. Thirdly, mice of the tumour-free sublines provided with C3H virus usually grow tumours that are not pregnancy-dependent but grow mostly pregnancy-dependent tumours when provided with virus from the tumour-prone BR6 mice. These results suggest that the BR6 virus and the C3H virus have different properties and that pregnancy-dependence in tumour growth is a result of both the type of virus and of its environment.

### COMMENT

Medical research is sometimes criticized for studying disease rather then health. P.C.W. certainly merited such criticism when he first became aware that a tumour-free subline was swamping the precious tumour-bearing strain. His immediate reaction, immediately carried out, was to kill all mice of the offending subline. His next reaction was a horrified realization of what he had done. Fortunately other tumour-free sublines arose and the mistake could be rectified. The question why the mice in these sublines do not develop tumours is obviously as, or more, interesting than the question why the mice of the parent strain do develop tumours.

The occurrence of the sublines was as unexpected as it is unexplained and we do not propose to speculate on its significance until more facts are available.

### SUMMARY

A colony of BR6 mice, derived from that established by Foulds (1947), has been maintained by brother-sister mating for 40 generations.

The incidence of mammary tumours in the 545 females whose mothers had tumours and who themselves had two or more litters was 95 per cent.

Five of the 29 mice comprising the tumour-free 5 per cent, when mated with their brothers, gave rise to tumour-free sublines.

One of these sublines has been maintained for 25 generations separated from the parent strain.

Skin can be grafted from parent strain into subline but tumours are only exceptionally transplantable in the same way.

We are very grateful to Messrs. L. E. G. Miall, B. F. Wise and D. E. Brown for looking after the colony and keeping the tumour records.

### REFERENCES

FOULDS, L.—(1947) *Brit. J. Cancer*, **1**, 362.—(1949*a*) *Ibid.*, **3**, 230.—(1949*b*) *Ibid.*, **3**, 240.—(1949*c*) *Ibid.*, **3**, 345.—(1954) *Cancer Res.*, **14**, 327.—(1956) *J. nat. Cancer Inst.*, **17**, 701.